# Facelift incision and superficial musculoaponeurotic system advancement in parotidectomy: case reports

**DOI:** 10.1186/s40902-015-0040-2

**Published:** 2015-11-02

**Authors:** Il-Kyu Kim, Hyun-Woo Cho, Hyun-Young Cho, Ji-Hoon Seo, Dong-Hwan Lee, Seung-Hoon Park

**Affiliations:** 1 Department of Oral and Maxillofacial Surgery, College of Medicine, Inha University, #7-206, 3rd St. Shinheung-dong, Choong-gu, Incheon 400-711 Korea; 2 Department of Oral and Maxillofacial Surgery, International St. Mary’s Hospital, Catholic Kwandong University College of Medicine, Incheon, Korea

**Keywords:** Parotidectomy, Facelift incision, SMAS flap, SCM muscle flap, Collagen implant

## Abstract

Surgical procedures for parotidectomy had been developed to gain adequate approach, prevent morbidity of nerve, and give esthetic satisfaction.

We performed two cases of parotidectomy through facelift incision. One case was reconstructed with superficial musculoaponeurotic system (SMAS) flap and sternocleidomastoid (SCM) muscle rotated flap at the parotid bed. In second case, same procedures were performed, but collagen membrane was additionally implanted for prevention of Frey’s syndrome. After surgery, two cases showed esthetic results without neck scar and hollow defect on parotid bed area.

## Background

**Fig. 1:**
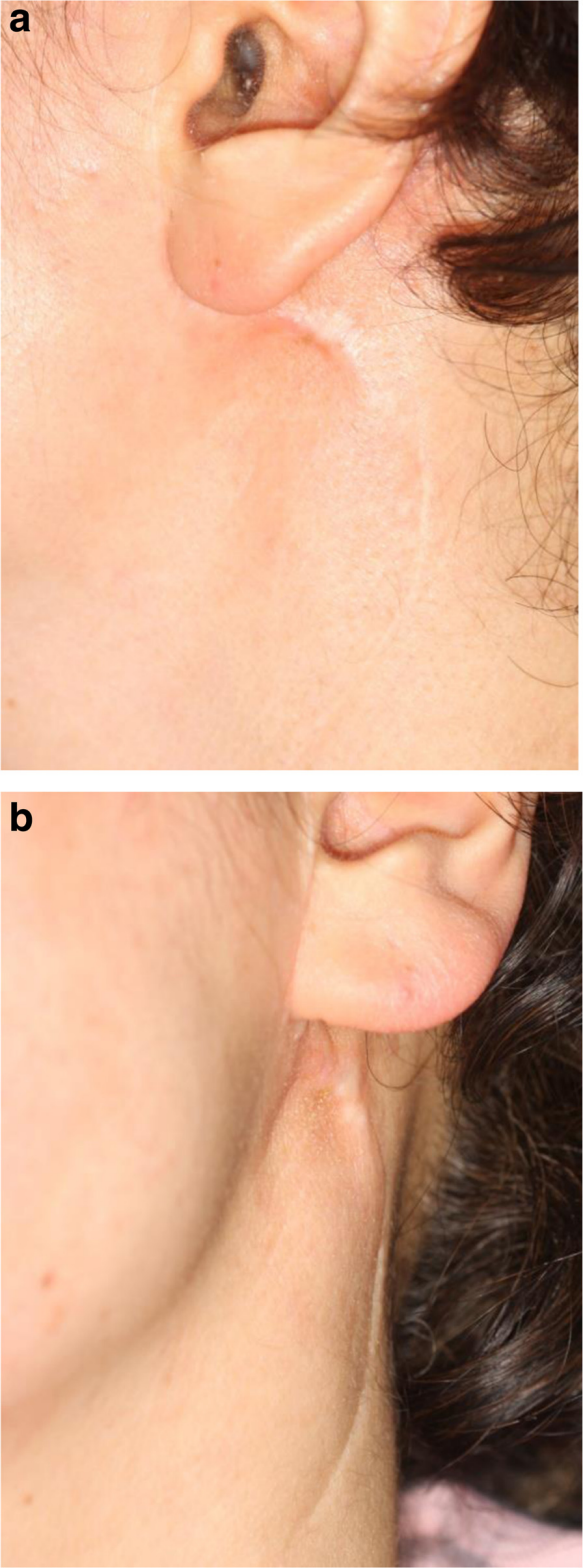
Example of the Blair incision. The patient received parotidectomy with modified Blair incision shows hypertrophic scar on neck (**a**) and depression on mandibular angle area (**b**)

Various approaches for resection of the parotid gland have been developed over the past 100 years. Several designs of incision for parotidectomy are possible. Ever since Gutierrez [1] introduced a guideline of incision for approaching the parotid gland, the surgical techniques for parotidectomy have been greatly advanced. Ideal incision line for resection of parotid gland should provide the wide field of operation and minimize the post-operative scars on the face and neck. Although the modified Blair incision can provide a proper operation sight for parotidectomy, downward neck incision from the ear lobe may give unwanted scars on the neck (Fig. 1a).

Recently, facelift incisions have often been used for surgery of the benign parotid tumor [2, 3]. It gives patients an esthetic satisfaction because the incision line can be hidden naturally in the auriculomastoid groove and hair line. This incision generally used in rhytidectomy also offers a large field of operation including a superior portion of sternocleidomastoid (SCM) muscle and lateral head and neck, as well as the parotid bed. This is helpful in reconstructing the parotid bed after partial or total parotidectomy. Unless the parotid bed is reconstructed, hollow space can be seen in the mandibular angle region which causes unaesthetic asymmetry (Fig. 1b).

The most common late complication after the parotidectomy is Frey’s syndrome. It is considered to be a misdirected growth of sectioned auriculotemporal nerve fibers and/or parasympathetic nerve fibers to the sweat glands of the skin overlying the parotid gland. The reported incidence of this syndrome is 2 to 80 % depending on the methods of reconstruction on the hollow parotid bed and the time-interval from the surgery [4].

The two cases of parotidectomy with facelift incision and reconstruction of superficial musculoaponeurotic system (SMAS) and SCM flap were performed and evaluated the esthetic and functional results.

## Case presentation

### Case 1

**Fig. 2:**
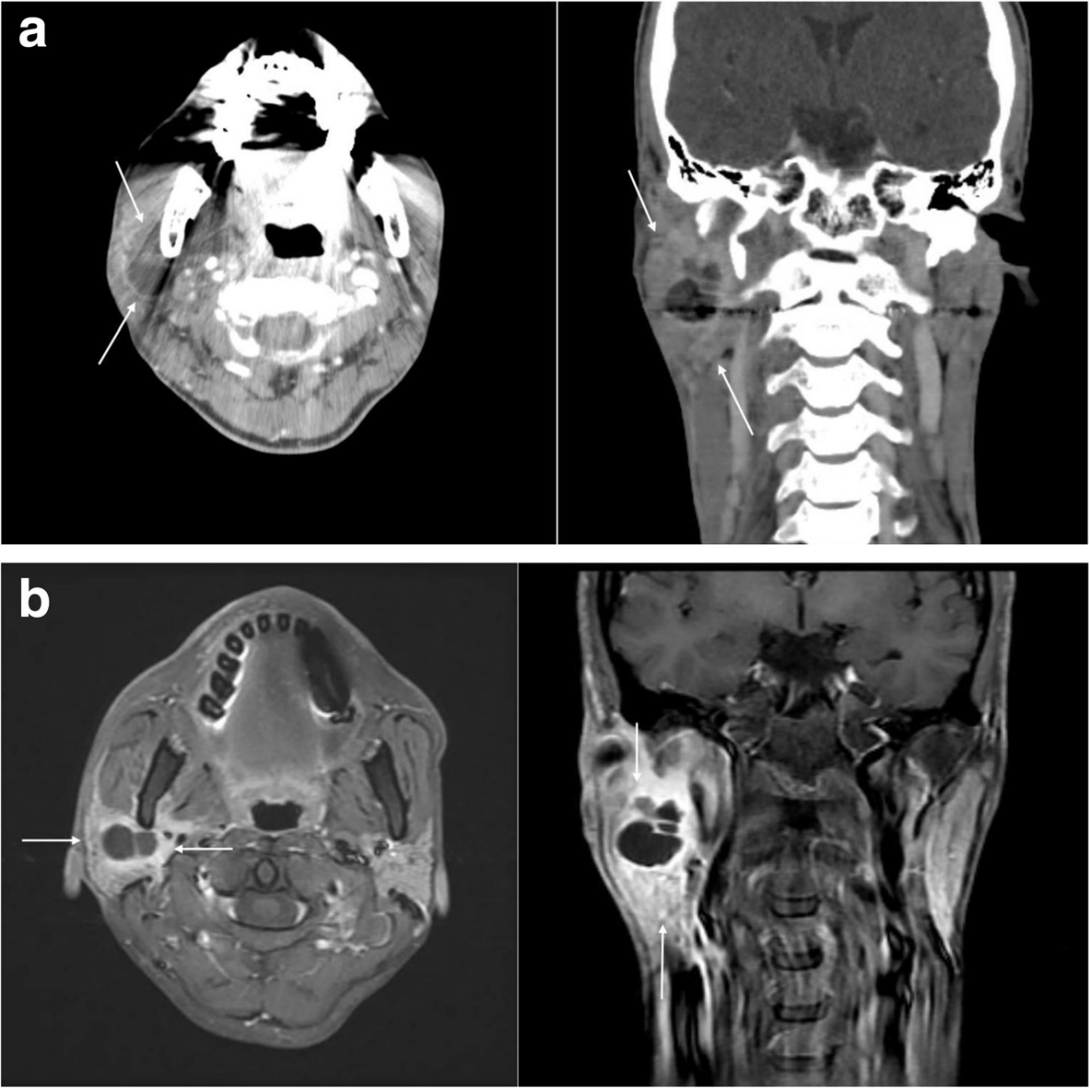
Pre-operative radiographic images of the case 1 patient. Parotid CT (**a**) and MRI (**b**) showed glandular enhancement with lobulating cystic lesions in the right parotid gland

The patient was a 47-year-old male whose chief complaints included the recurrent swelling and tenderness of the mandible angle area as well as fever and trismus for 3–4 months. The pre-operative parotid computed tomography (CT) and magnetic resonance imaging (MRI) showed 2.6 × 1.5 × 2.8 cm sized lobulating septated cystic lesion in the right parotid gland and diffused glandular enhancement and enlargement with acute/chronic inflammation (Fig. 2a, b). Mild diffuse narrowing of right proximal main duct was noted in sialography. The pre-operative diagnosis was 2.6 × 1.5 × 2.8 cm sized salivary gland cyst in the right parotid gland with acute/chronic inflammation. Afterwards, total parotidectomy with rotational SCM flap and advancement of SMAS flap under the general anesthesia was conducted.

**Fig. 3:**
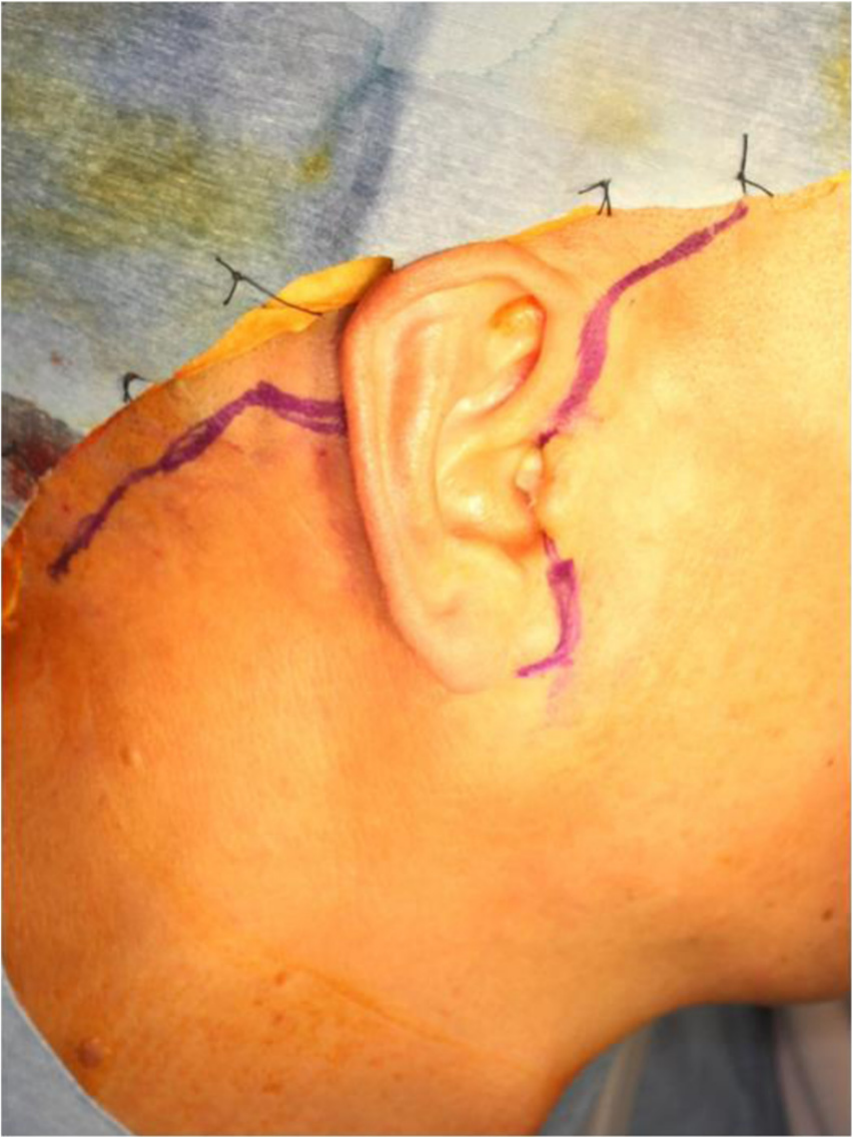
Facelift incision line of the case 1 patient. Incision line of facelift which is hidden on postauricular groove and hairline

The incision line was continuous, running from the temporal area to the preauricle, postauricle, and finishing on the hairline (Fig. 3). The inverted hockey stick incision at temporal area began in the posterior-inferior direction at a 45° downward angle for 3 cm, running to the superior portion of the ear. Then, it ran behind the tragus and followed to the earlobe fold and the auriculomastoid groove up to the upper 1/3 of the ear. After that, the incision line was naturally curved to the hairline (Fig. 3).

**Fig. 4:**
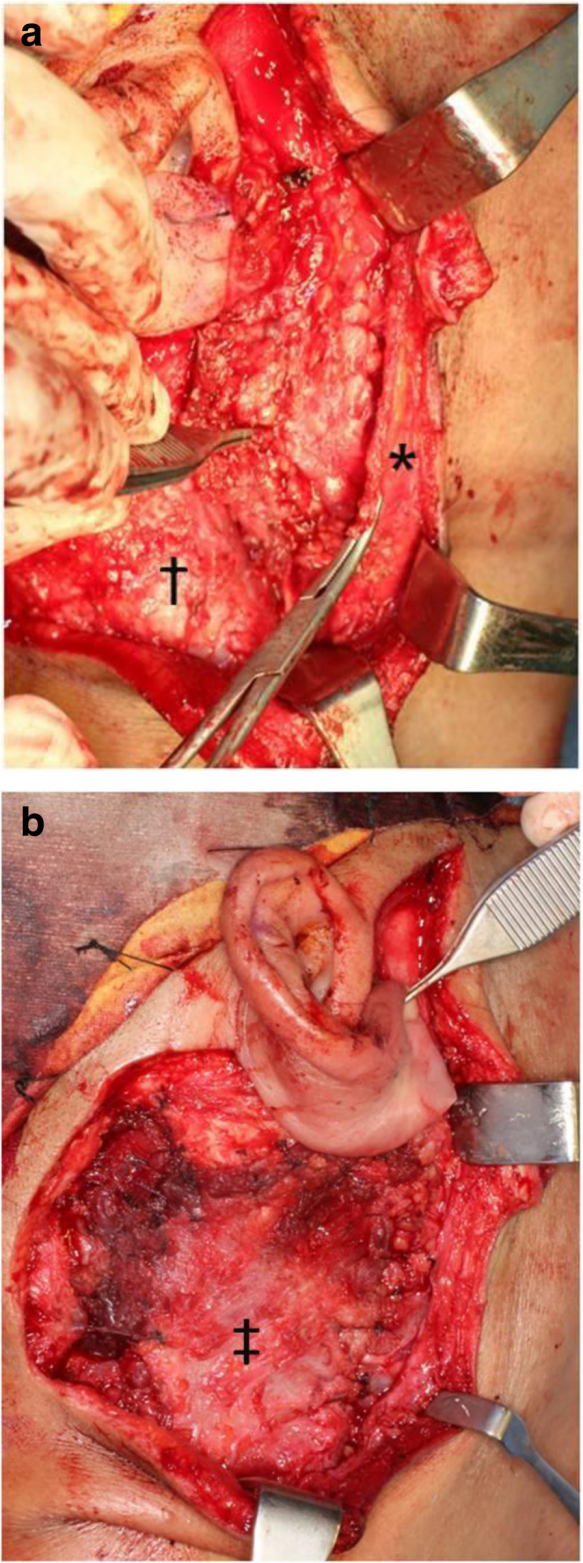
SMAS flap and SCM muscle flap procedure of the case 1 patient. SCM muscle (†) before rotation was shown below the parotid gland (**a**). SMAS flap (*) was dissected between skin flap and parotid gland (**a**) and sutured with rotated SCM muscle flap (‡) (**b**)

The skin flap was raised through the subcutaneous dissection at postauricular region as a full-thickness flap. The extensive dissection was made on temporal and zygomatic region to separate the attachments between SMAS and the skin. SMAS layer was seen cephalic from platysma layer after dissection of skin flap. At this level, transverse incision was made on inferior border of zygomatic arch. And then a vertical incision was made from preauricular region to the posterior border of the platysma. SMAS layer was raised from the parotid fascia (Fig. 4a). Grayish color of parotid gland was distinct from the yellowish color of SMAS layer.

Parotidectomy was conventionally performed with the dissection of the facial nerve trunk located around the mastoid process. During this procedure, meticulous blunt dissection was needed. The great auricular nerve was not preserved.

After the superficial and deep lobe of the parotid gland was successfully resected, a hollow space was covered with two layers. A superficial layer of SCM muscle was stripped off from the mastoid process and rotated anteriorly to the parotid bed. Then, the SMAS flap was advanced to the mandible angle and sutured with SCM muscle flap (Fig. 4b).

**Fig. 5:**
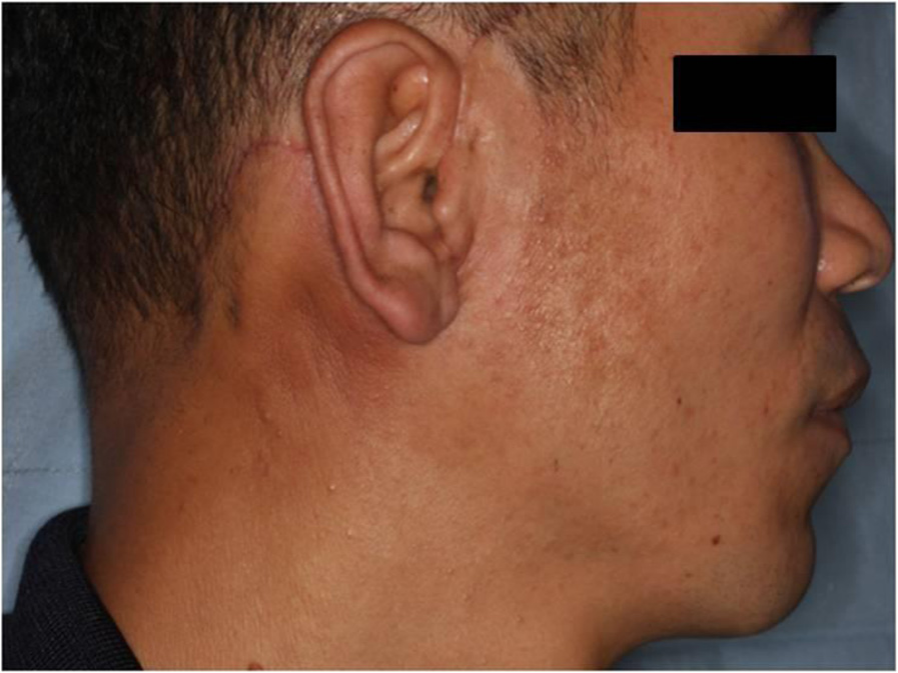
Post-operative clinical image of the case 1 patient (1 month after operation). The patient showed relatively unnoticeable scars (especially no scar on neck). The incision line can be naturally covered by hair

At the follow-up of 2.5 months, the patient exhibited adequate function of facial expression and facial contour of the mandible angle. Also, it is difficult to find scarring on the preauricular and neck area (Fig. 5). As of yet, there were no symptoms of Frey’s syndrome.

### Case 2

**Fig. 6:**
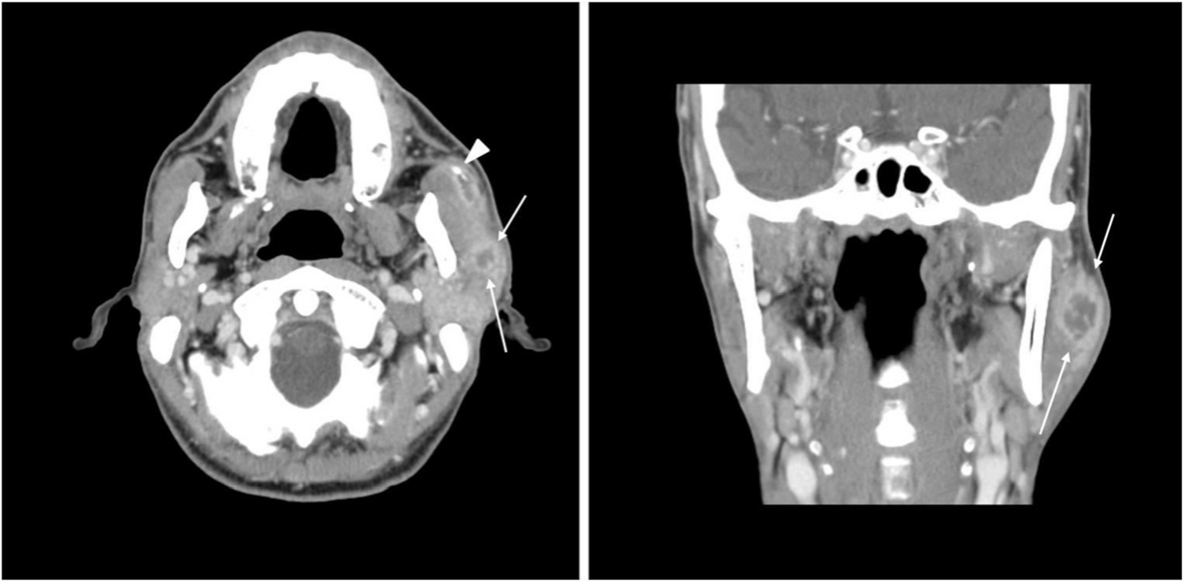
Pre-operative mandible CT of the case 2 patient on first visit. Mandible CT showing salivary stone (*arrow head*) and acute/chronic suppurative sialoadenitis with irregular thick-walled, septated cystic mass (*arrow*) in superficial lobe of right parotid gland

**Fig. 7:**
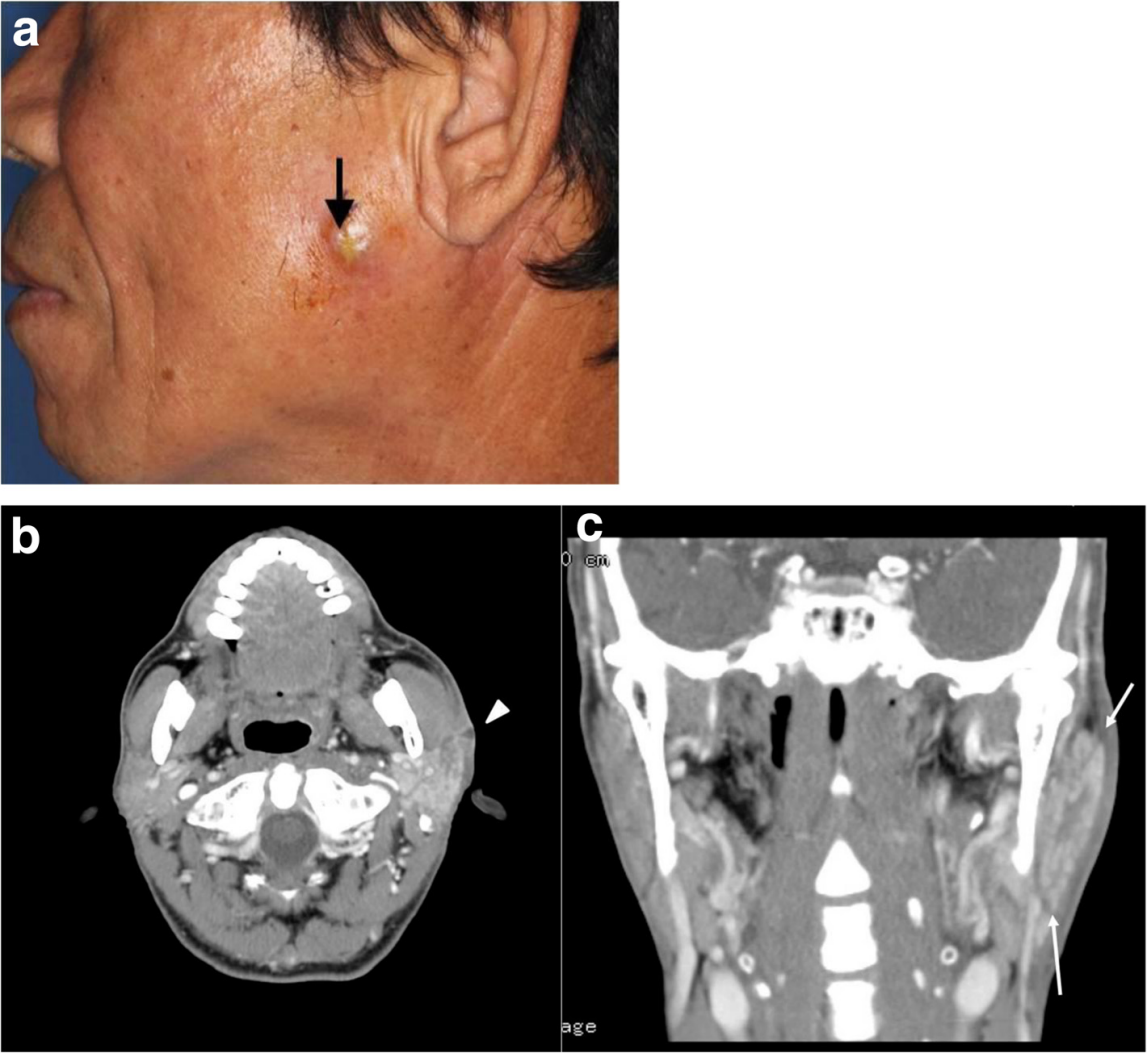
Clinical and mandible CT images of the case 2 patient (3 months after first visit). Cutaneous fistula was shown on the skin of right cheek (**a**). The fistula (**b**, *arrow head*) with enlarged enhanced mass (**c**, *arrow*) was found on mandible CT

The patient was a 59-year-old male who complained swelling and tenderness on the right cheek. Mandible CT with contrast showed 0.9 cm sialolithiasis and acute suppurative sialoadenitis with 1.8 cm irregular thick-walled, septated cystic mass in superficial lobe of the right parotid gland (Fig. 6). The patient refused operation for his personal reasons, but he visited our office 3 months later with cutaneous fistula on the skin of the right cheek (Fig. 7a). Acute/chronic parotid abscess with fistular tract and cellulitis due to sialolith was shown in mandible CT (Fig. 7b, c).

**Fig. 8:**
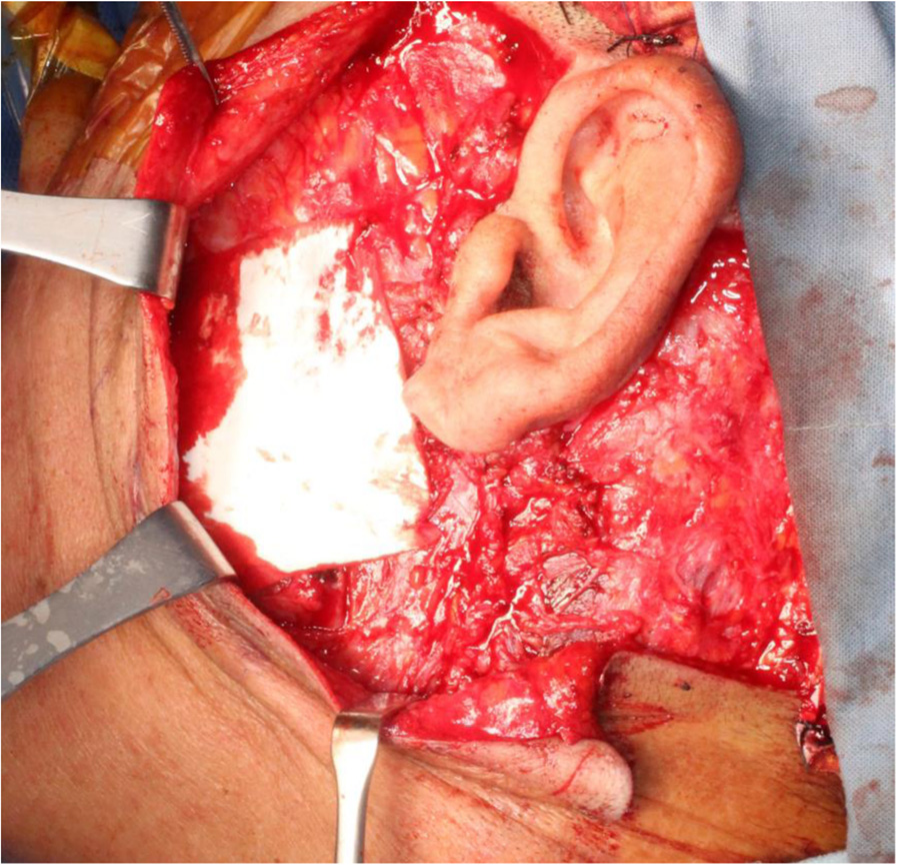
SMAS flap and SCM muscle flap procedure of the case 2 patient. The parotid bed was reconstructed with combination of SMAS flap and SCM flap with 4 × 5 cm collagen implant

Superficial parotidectomy under the general anesthesia was planned. Facelift incision and dissection of SMAS flap were conducted in the same way with the former case. After resection of superficial lobe of the parotid gland, SMAS flap was rotated, advanced over the parotid bed, and sutured to anteriorly rotated SCM muscle flap. And 4 × 5 cm collagen implant (Lyoplant™, B. Braun, Tuttlingen, Germany) was additionally placed above the parotid bed for reinforcement of prevention of Frey’s syndrome (Fig. 8).

**Fig. 9:**
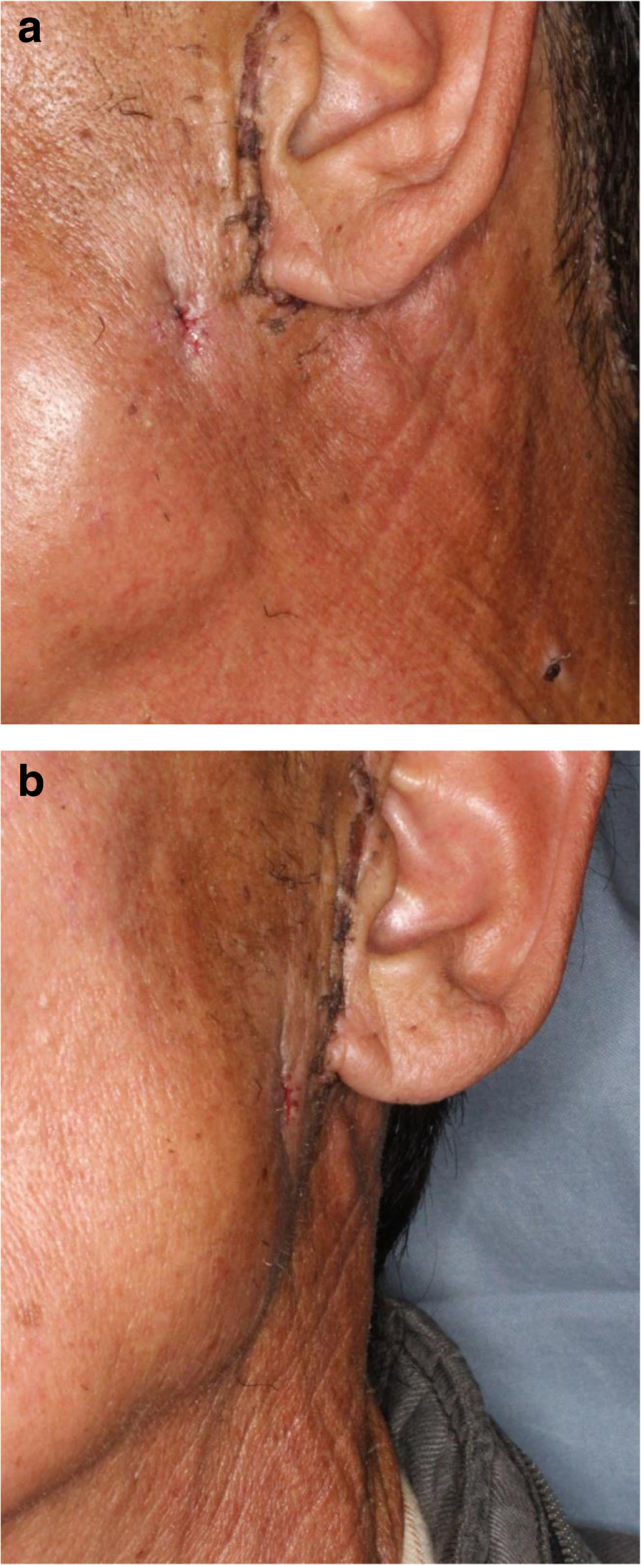
Post-operative images of the case 2 patient (2 weeks after operation). Patient showed no visible scar on neck (**a**) and no hollow defect on parotid bed (**b**)

After surgery, the patient showed no neck scarring, hollow defect, and Frey’s syndrome (Fig. 9).

### Discussion

There are two major complications in dealing with the parotidectomy. There are the functional problems which are associated with morbidity of the facial nerve and Frey’s syndrome. Another is esthetic problems such as neck scarring and hollow space of the parotid region which affects the post-operative social life of the patient.

In an esthetic point of view, visible scars on the face and neck after surgery can negatively impact on an individual’s quality of life. There were many attempts to modify the Blair incision in order to avoid scarring on the neck. Nouraei et al. [5] showed in an anatomical study that facelift incisions provide generous access to all regions of the parotid gland, which was similar to Blair’s incision. Lee et al. [6] compared facelift incision with modified Blair incision in parotidectomy of benign lesion without reconstruction. The mean scar satisfaction score was significantly higher in facelift incision group. Bianchi et al. [7] reported that facelift incision alone in partial parotidectomy led to a high statistically significant improvement in the esthetic outcome. Facelift incisions allow the incision lines to be hidden in the postauricular region and hairline which provide improved satisfaction for the patient. It can also provide easier approach to SCM muscle [8].

Cesteleyn et al. [9] reported that the incidence of Frey’s syndrome was reduced from 33 to 4 % in cases using musculoaponeurotic layer. Allison and Rappaport [9] reported only two cases of Frey’s syndrome in 112 patients who had undergone operation with a SMAS flap. This SMAS flap is also useful to augment hollow defect [3, 9, 10]. The dissection of SMAS flap is easy and rapid and was conducted on the parotid region which is time-saving. It functions as a membrane for guided tissue regeneration. The hematoma below the SMAS flap may become to scar tissue and fill the parotid bed.

Sood et al. [11] reported that SCM muscle flap reduced the incidence of Frey’s syndrome. Two of 11(18.2 %) patients from the group which had a SCM rotation flap showed evidence of gustatory sweating. However, Gooden et al. [12] reported no statistical difference in incidence rate of Frey’s syndrome between the group reconstructed with SCM rotation flap and control group which had not undergone reconstruction. Effectiveness of SCM muscle flap in preventing Frey’s syndrome is controversial [13]. Nevertheless, SCM muscle flap have other benefits unlike other procedures such as the temporoparietal or platysma muscle flap. The SCM muscle flap is easy to rotate without an additional incision into the parotid bed. And SCM muscle flap provides an adequate volume to fill the hollow space [11, 12]. Also, there is a low risk of flap necrosis because of abundant vascularization.

After parotidectomy, SMAS flap is too small to cover all defects of the parotid bed. This may lead to unsatisfactory esthetic results to patients. Combinations of various procedures with SMAS flap can be performed to increase an esthetic satisfaction. Zhao et al. [14] reported that the sub-SMAS flap combined with the SCM muscle flap provided more improved esthetic results than the sub-SMAS flap alone. Chen et al. [10] used SMAS fold flap with allograft dermal matrix (ADM) to repair the parotid bed. This showed a statistical difference in rates of satisfaction of facial contour between SMAS only group (91.9 %) and SMAS with ADM group (100 %).

## Conclusions

In this study, we performed two cases of parotidectomy through facelift incision. One case was reconstructed with superficial musculoaponeurotic system (SMAS) flap and Sternocleidomastoid (SCM) muscle rotated flap at the parotid bed. In second case, same procedures were performed, but collagen membrane was additionally implanted for prevention of Frey’s syndrome. After surgery, two cases showed esthetic results without neck scar and hollow defect on parotid bed area.

## Consent

Written informed consent was obtained from the patients for publication of this case report and any accompanying images. A copy of the written consent is available for review by the Editor-in-Chief of this journal.
